# Serum proteomic analysis focused on fibrosis in patients with hepatitis C virus infection

**DOI:** 10.1186/1479-5876-5-33

**Published:** 2007-07-11

**Authors:** Ian R White, Keyur Patel, William T Symonds, Anouk Dev, Philip Griffin, Nikos Tsokanas, Mark Skehel, Chiang Liu, Amany Zekry, Paul Cutler, Mahanandeeshwar Gattu, Don C Rockey, Michelle M Berrey, John G McHutchison

**Affiliations:** 1 Department of Disease and Biomarker Proteomics, GlaxoSmithKline Pharmaceuticals, Stevenage, UK; 2 Division of Gastroenterology, Duke Clinical Research Institute, Duke University Medical Center, Durham, North Carolina, USA; 3 Division of Clinical Pharmacology and Discovery Medicine, GlaxoSmithKline Pharmaceuticals, Research Triangle Park, North Carolina, USA; 4 Infectious Diseases Centre of Excellence for Drug Discovery, Virology, GlaxoSmithKline Pharmaceuticals, Stevenage, UK; 5 Molecular Discovery Research-Informatics, GlaxoSmithKline Pharmaceuticals, Research Triangle Park, North Carolina, USA; 6 Department of Digestive and Liver Diseases, University of Texas Southwestern, Dallas, Texas, USA

## Abstract

**Background:**

Despite its widespread use to assess fibrosis, liver biopsy has several important drawbacks, including that is it semi-quantitative, invasive, and limited by sampling and observer variability. Non-invasive serum biomarkers may more accurately reflect the fibrogenetic process. To identify potential biomarkers of fibrosis, we compared serum protein expression profiles in patients with chronic hepatitis C (CHC) virus infection and fibrosis.

**Methods:**

Twenty-one patients with no or mild fibrosis (METAVIR stage F0, F1) and 23 with advanced fibrosis (F3, F4) were retrospectively identified from a pedigreed database of 1600 CHC patients. All samples were carefully phenotyped and matched for age, gender, race, body mass index, genotype, duration of infection, alcohol use, and viral load. Expression profiling was performed in a blinded fashion using a 2D polyacrylamide gel electrophoresis/LC-MS/MS platform. Partial least squares discriminant analysis and likelihood ratio statistics were used to rank individual differences in protein expression between the 2 groups.

**Results:**

Seven individual protein spots were identified as either significantly increased (α_2_-macroglobulin, haptoglobin, albumin) or decreased (complement C-4, serum retinol binding protein, apolipoprotein A-1, and two isoforms of apolipoprotein A-IV) with advanced fibrosis. Three individual proteins, haptoglobin, apolipoprotein A-1, and α_2_-macroglobulin, are included in existing non-invasive serum marker panels.

**Conclusion:**

Biomarkers identified through expression profiling may facilitate the development of more accurate marker algorithms to better quantitate hepatic fibrosis and monitor disease progression.

## Background

Hepatic fibrosis is the final common pathway of liver injury in most liver diseases and can lead to cirrhosis, which is responsible for most clinical sequelae. Assessment of the degree of fibrosis usually involves a liver biopsy. Although liver biopsy has long been considered to be the gold standard for assessing fibrosis, the procedure is invasive, potentially associated with important complications to the patient, and provides only a semi-quantitative assessment of fibrosis. Furthermore, the diagnostic accuracy of a biopsy in the staging of fibrosis is seriously affected by errors in sampling and intra-observer variation [1-5].

Non-invasive biomarkers may provide a more dynamic reflection of fibrogenesis; the identification and quantification of such markers could lead to the development of a non-invasive means to monitor disease progression [6,7]. Serum biomarker panels (eg FibroTest, BioPredictive, Paris, France) and transient elastography (FibroScan^®^, Echosens, Paris, France) methods have been validated in chronic hepatitis C (CHC) patients, and incorporated into clinical practice in many countries [8-10]. These non-invasive measures may also provide prognostic information that may be of clinical significance in CHC patients with advanced disease [11]. However, these methods are limited by variability among liver diseases of other etiology, and in their ability to accurately differentiate individual fibrosis stages [6]. Nevertheless, biomarkers and other non-invasive approaches remain promising alternatives to biopsy for monitoring disease progression or assessing the efficacy of antifibrotic therapies.

We hypothesized that elucidating novel proteins differentially expressed in carefully pedigreed patients with advanced fibrosis versus those with minimal fibrosis would allow development of novel assays or a profile that could be used for accurate staging of hepatic fibrosis. We thus analyzed protein expression levels in serum samples from patients with liver fibrosis as a result of chronic hepatitis C virus infection.

## Methods

### Sample selection

Serum samples from 23 treatment-naïve chronic hepatitis C patients with no or mild fibrosis (METAVIR stage F0, F1) and 21 patients with advanced fibrosis (F3, F4) (Table 1) were retrospectively identified from a large hepatitis patient and clinical research database that includes demographic, virologic, serologic, and histologic data from more than 1600 individuals enrolled in treatment protocols since 1992. The repository was developed at the Scripps Clinic and Research Foundation and was moved to Duke University and continued from 2002. Patients enrolled in the database gave informed consent for the studies in which they participated and for the use of their data and stored serum in future studies. Such future use had also been approved separately by the institutional review boards at each institution. Serum samples were snap-frozen within 4 hours of collection and stored at -70°C. Samples were no more than 7 years old and had undergone a single freeze/thaw cycle. METAVIR score on liver biopsies with at least 6 portal tracts, performed at or near the time of serum sample collection, had been determined by 3 experienced pathologists (κ coefficient of agreement of 0.83). Mean biopsy (± SD) length was 13.1 ± 3.73 mm, and patients were matched for age, gender, race, body mass index, genotype, duration of infection, alcohol use, alanine aminotransferase level, necroinflammatory activity, and serum hepatitis C viral concentrations (Table 1).

**Table 1:** Patient Demographics Based on Fibrosis Staging.

	**METAVIR Stage**	
	
	**F0-F1**	**F3-F4**
**Age **(y)	43.8 ± 9.75	48.1 ± 4.18
**Sex **(M/F)	21/2	19/2
**Race**		
White	20	19
Hispanic	2	2
Asian	1	0
**HCV genotype**		
1	18	14
2	3	2
3	2	5
**Duration of infection **(y)	22.1 ± 6.03	22.9 ± 6.68
**ALT **(IU/mL)	101.7 ± 61.64^a^	129.8 ± 69.19^b^
**METAVIR Activity**	1 (0–3)^c^	2 (0–3)^d^

**HCV RNA **(log_10 _copies/mL)	6.53 ± 0.39	6.43 ± 0.43

Results shown as mean ± standard deviation, number, or median (range); ALT, alanine aminotransferase; HCV, hepatitis C virus; METAVIR Activity, histological score of necroinflammation based on interface, lobular, and portal inflammation. a vs. b, p = 0.16; c vs. d, p = 0.07

### Two-dimensional polyacrylamide gel electrophoresis

Frozen serum samples were thawed and centrifuged at 13,000 × g for 10 min. Albumin depletion of the supernatants was immediately carried out using Mimetic Blue SA affinity chromatography (Prometic Biosciences Ltd., Cambridge, UK). The depleted material was concentrated using Vivaspin 2 (Sartorius Ltd., Epsom, UK) centrifugal filtration devices (5000 Da MWCO) and diluted into isoelectric focusing sample buffer (8 M urea, 4% [w/v] CHAPS, 100 mM DTT, 0.8% carrier ampholytes pH 3–10 NL). Duplicate samples of 200 μg protein were subjected to isoelectric focusing on 24-cm, immobilized, non-linear, pH 3–10, ReadyStrips (BioRad Laboratories, Hemel Hempstead, UK) for 64,000 Vh using Multiphor electrophoresis apparatus (GE Healthcare, Little Chalfont, UK). Focused strips were subjected to reduction and alkylation in the presence of excess SDS using DTT and then iodoacetamide, followed by SDS-PAGE on 12% (w/v) acrylamide slab gels (20 × 25 × 0.15 cm), essentially as described by Görg and coworkers [12]. Proteins were stained with SYPRO Ruby (Invitrogen Ltd., Paisley, UK), and 16-bit digital images were acquired on FUJI FLA5000 imaging systems, employing excitation at 473 nm with a 575 nm LP filter.

### Image analysis and spot selection

Individual protein features were detected, matched, and quantified using Progenesis Discovery software (Nonlinear Dynamics, Newcastle-upon-Tyne, UK). Principal Components Analysis (PCA) [13] was carried out on the exported dataset using Simca-P+ (Umetrics, Umea, Sweden), and all matched spots were ranked based on the magnitude of expression change between the 2 groups with mild or advanced fibrosis using partial least squares discriminant analysis (PLS-DA) [13] and likelihood ratio statistics [14]. The latter method is designed specifically for proteomic data and ensures appropriate weighting is given to the data independent of any missing values. Using PLS-DA, the most important protein spots were identified using the variable important plot list.

### Protein identification

Protein identifications were carried out essentially as described previously [15]. Briefly, polyacrylamide gel cores of protein spots with altered expression profiles were excised using a KCore automated cutter (KBiosystems, UK). The samples were then subjected to in situ enzymatic digestion using a MassPrep liquid handling system (Waters, UK). The digests were analysed by nanoscale capillary LC/MS/MS using a Waters CapLC and Stream Select Module to deliver a flow of 5 μL/min, split to approximately 200 nL/min. An LC Packings u-Precolumn, C18 PepMap 100 (Dionex, The Netherlands) guard column trapped the peptides prior to separation on a NAN75-15-03-C18-PM, PepMap 100 column, 75 μm ID × 15 cm. Peptides were back-flushed from the guard column onto the analytical column at 200 nL/min and eluted with a gradient of acetonitrile. Mass spectrometric information was obtained using an orthogonal acceleration quadrupole-Tof mass spectrometer (Q-Tof Ultima API, Waters, UK) equipped with a Z-spray source for nanoflow analysis and operated in V-mode. Data directed analysis was carried out, where automatic MS/MS was acquired on the 8 most intense, multiply charged precursor ions in the m/z range 400–1500. MS/MS data were acquired over the m/z range 50–1975. LC/MS/MS data were then searched against an in-house non-redundant protein database using the Mascot search engine program (Matrix Science, UK) [16].

## Results and Discussion

### Data visualization by Principal Components Analysis (PCA)

**Figure 1:**
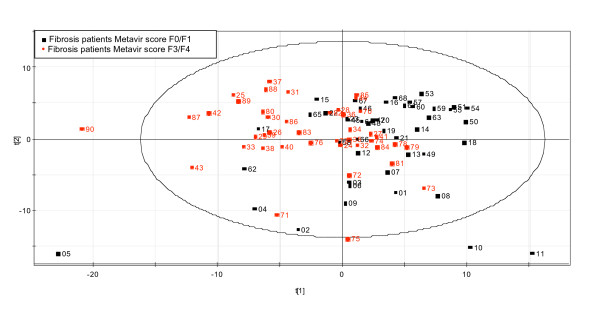
Principal Components Analysis (PCA) of Two-dimensional Polyacrylamide Gel Electrophoresis Data. Gel images were processed using a commercially available analysis package followed by export into Microsoft Excel, normalization, log transformation, and PCA using Simca P+.

The scores plot from the PCA analysis (Figure 1) showed evidence of some separation between the mild and advanced fibrosis groups in the first principal component, which is indicative of variation in their respective protein expression profiles. Despite the quality of this highly pedigreed sample set, the clustering of the 2 sample sets is not complete, reflecting the biological variability inherent in clinical patient cohorts.

### Spot selection

**Figure 2:**
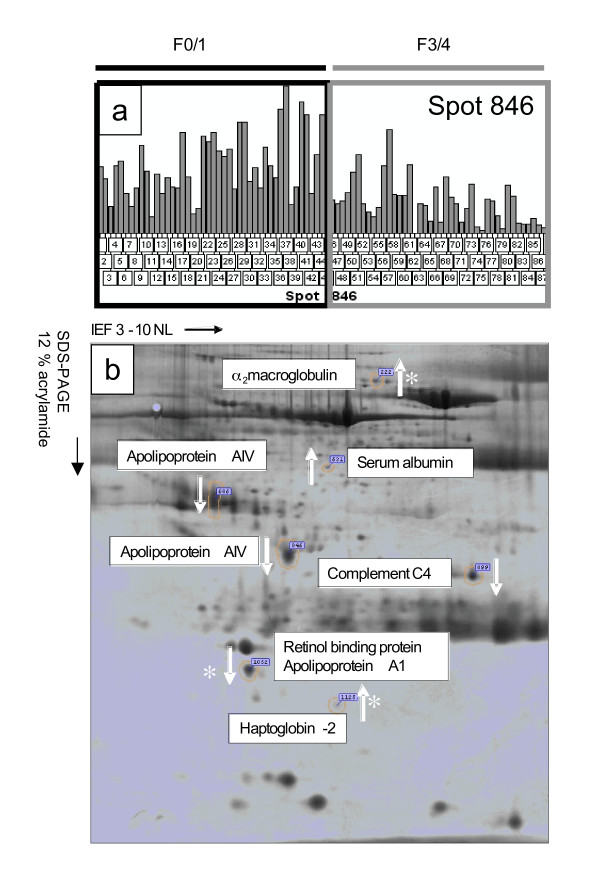
Spot Selection and Two-dimensional Map of Putative biomarkers. (a) Image analysis output from Progenesis showing the relative volume (pixel intensity * mm^2^) of smaller variant of ApoA4 (spot 846) across all experimental gels. Gel 52 was excluded from analysis due to aberrant isoelectric focusing. (b) Image of 2D PAGE showing positions of putative markers. The direction of change (advanced relative to moderate disease) is indicated by the white arrows, and the asterisk signifies proteins whose behavior is consistent with the commercially available FibroTest. Expression of albumin reflects fragments not adsorbed by affinity-based mimetic ligands.

Seven protein spots with altered expression levels between mild and advanced fibrosis patients were identified (Figure 2). Compared with patients with mild fibrosis, patients with advanced disease had increased serum expression of 3 spots, which were identified as α_2_-macroglobulin (A2M), haptoglobin, and a fragment of albumin. Conversely, the expression of 4 spots was decreased: serum retinol binding protein (SRBP), complement C-4, apolipoprotein A-1 (ApoA1), and 2 variants of apolipoprotein A-IV (ApoA4). ApoA1 and SRBP were identified in the same spot, and it was not possible to determine which protein contributed to the alteration in intensity (Figure 2).

While haptoglobin, A2M, and ApoA1 are well-established markers of hepatic fibrosis, SRBP, complement C4A, and ApoA4 are potentially novel biomarkers of disease.

### Protein variants

As well as offering a route to detecting subtle changes in protein structure, including post translational modifications, two-dimensional polyacrylamide gel electrophoresis enables observation of events such as protein processing and variations in gene splicing. For a number of proteins studied in this analysis, the electrophoretic migration and the peptide mass spectra used to assign protein identity provide insight into the nature of the individual protein variant on the gels.

#### α2 macroglobulin

In this study, the migration of the spot was inconsistent with the presence of intact A2M (163.1 kDa) molecule. The peptides identified by mass spectrometry, which were also used to assign identity, are exclusively derived from the N-terminal half of the molecule, suggesting increased processing of the intact molecule may be occurring in the advanced disease state (see addtional file 1).

#### Complement C4 precursor

Complement C4 (192.6 kDa, pI 6.65) is a central component of the classical complement pathway. Like A2M, the position of the protein spot on the gels (~30 kDa, pI~8) is inconsistent with migration of intact protein. Prior to secretion, the intact molecule is cleaved into distinct α, β, and γ chains. To identify this protein, 3 peptides were mapped back to the database sequence using mass spectrometry, and all of them were located in the C-terminal, γ chain region, suggesting that in advanced disease net production of this subunit is downregulated (addtional file 1, part b).

#### Apolipoprotein A-IV

ApoA4 was identified in 2 spots that appeared to be downregulated in the advanced fibrosis patients. The location of 1 of these variants is consistent with migration of the intact molecule, whereas the second more basic, smaller form suggests differential processing of ApoA4 in advanced disease (addtional file 1, part c). Downregulation of ApoA4 has been reported at the gene level in a thioacetamide-induced rat model of hepatic fibrosis [17]. At the protein level, a small acidic fragment of the protein was detected in the serum of individuals with hepatitis B infection [18]. Although the fragment was distinct from the variants described here, it was down-regulated in infected individuals and could be correlated with necroinflammatory scores.

#### SRBP and ApoA1

One spot identified with altered expression contained 2 proteins, SRBP and ApoA1. Downregulation of ApoA1 is known to correlate with disease progression, and the spot was close to the documented location of ApoA1 but was also consistent with migration of SRBP [19,20]. As the mass spectrometric approach used for protein identification in this study did not enable quantification of individual proteins in gel spots, it is not possible to say which of these proteins has contributed to the alteration in intensity without orthogonal validation studies.

### Monitoring fibrosis progression

While this was an exploratory study with a small number of patients, the identified protein isoforms associated with fibrosis offer insight into the pathology of fibrosis and provide possibilities for new and more selective bioassays. Despite the small sample size, the identification of haptoglobin, A2M, and ApoA1 in this study provides validation for the proprietary FibroTest (BioPredictive, Paris, France), which is available in several countries as a non-invasive alternative to biopsy [21]. Although low serum haptoglobin may be seen in advanced cirrhosis and hemolysis, increased expression of haptoglobin in these patients likely reflects its role as an acute phase reactant to an inflammatory disease in our cohort of patients with preserved hepatic function. As albumin detection could have been affected by impaired hepatic synthetic function in decompensated cirrhotic disease, such patients were excluded in this study. Several other specific extracellular matrix markers, including hyaluronic acid [22-24], tissue inhibitor of metalloproteinases-1 (TIMP-1) [23,24], matrix metalloproteinase (MMP)-1, MMP-2, MMP-9, and procollagen type III N-terminal peptide (PIIINP) [22,23] have also recently been identified as noninvasive markers of fibrosis. Further studies are underway to explore whether these newer markers can be validated as predictive in larger cohorts of patients, and to assess their utility in following changes in fibrosis over time. However, a significant limitation of current serum fibrosis markers is their ability to only differentiate between mild and advanced disease and not individual fibrosis stages. This relates in part to the limited accuracy of a liver biopsy for the diagnosis of individual fibrosis stage that may be due to inherent issues with sampling error for non-homogenously distributed disease, and observer differences in biopsy interpretation. These limitations in our "gold standard" may also reduce our ability to develop more accurate predictive biomarkers.

A limitation of our exploratory study was the absence of comparative protein expression profiles from healthy control sera or other chronic liver diseases. However, our aim was to answer a relatively simple, clinically relevant question using this open proteomics platform; the ability to differentiate advanced from mild stage disease in a cohort of hepatitis C patients matched for host and viral factors that could influence fibrosis progression. Secondary studies of validated biomarkers using robust platforms may be able to evaluate differential protein expression between fibrosis stages or other chronic liver disease. Any future diagnostics would need to be optimized for reproducibility and ease of use. Cost and availability of an assay would also affect clinical utility. In addition, the study of proteomics has inherent limitations. Several gel and non-gel-based multidimensional separation methods to explore the peptidome can be utilized. The 2-D gel methodology in this study is labor intensive, has a lower detection limit of 12 kDa., and is unable to differentiate hydrophobic or low abundance proteins. However, it represents an established and rapid method for targeting differential protein expression. Although matrix-assisted (MALDI) and surface-enhanced laser desorption/ionization (SELDI) mass spectrometry techniques are relatively newer approaches for protein detection, highly sensitive, and useful for complex biological samples, there are inherent issues with sample preparation and reproducibility [25]. Also, proteins are not directly visualized with chromatographic retentate methods such as SELDI, and instead rely on multifactorial bioinformatics for peak comparisons and data analysis. Future diagnostics could incorporate metabolomic changes seen during fibrosis progression as well. This could potentially improve diagnostic accuracy in relation to fibrogenesis, provide prognostic information, and help to integrate targeted non-invasive approaches into the clinical setting.

## Conclusion

Using protein expression profiling of hepatitis C patients, we identified 7 protein spots that were associated with fibrosis progression. While 3 of the markers are components of the commercially available FibroTest, the remaining 4 may facilitate the development of more sensitive and specific assays to monitor fibrosis progression.

## Abbreviations

Apolipoprotein A-1, ApoA1; apolipoprotein A-IV, ApoA4; α_2_-macroglobulin, A2M; Principal Components Analysis, PCA; serum retinol binding protein, SRBP.

## Competing interests

Ian White, William Symonds, Philip Griffin, Nikos Tsokanas, Mark Skehel, Chiang Liu, Paul Cutler, Mahanandeeshwar Gattu, and Michelle Berry are current or former employees of GlaxoSmithKline, which provided funding for aspects of this study. Keyur Patel and John McHutchison have served on advisory boards for and received research support from GlaxoSmithKline. The GI/Hepatology Research Division at Duke University Medical Center has a funded academic alliance with GlaxoSmithKline.

## Authors' contributions

IW, PG, NT, and JMS performed electrophoresis, image analysis, and protein identification; NT, MG, and CL performed the statistical and bioinformatics analysis; IW, KP, WS, AD, AZ, PC, DR, MB, and JGM were involved in conception and design of the study, selection and coordination of study samples, and drafting the manuscript; all authors read and approved the final manuscript.

## Supplementary Material

Additional file 1 Protein variants for α2macroglobulin, apolipoprotein A-IV, and Complement C4A. Peptides identified by LC-MS/MS, which were successfully mapped back to database entries, are entered in bold and are underlined. (a) *α2macroglobulin*. All peptides identified originate in the N-terminal region of the molecule. (b) *Complement C4A*. Location of peptides used to assign identity to C4A. The C-terminal γ-chain region is in grey text. (c) *Apolipoprotein A-IV*. Peptide coverage of the 2 variants identified indicating greater peptide coverage of the larger, more acidic molecule. Click here for file
