# Supplementary material for: Serum proteomic analysis focused on fibrosis in patients with hepatitis C virus infection
Source: J Transl Med. 2007 Jul 11;5:33. doi: 10.1186/1479-5876-5-33 (PMC1971245; doi:10.1186/1479-5876-5-33)
Supplement: Additional file 1 — Protein variants for α2macroglobulin, apolipoprotein A-IV, and Complement C4A. Peptides identified by LC-MS/MS, which were successfully mapped back to database entries, are entered in bold and are underlined. (a) α2macroglobulin. All peptides identified originate in the N-terminal region of the molecule. (b) Complement C4A. Location of peptides used to assign identity to C4A. The C-terminal γ-chain region is in grey text. (c) Apolipoprotein A-IV. Peptide coverage of the 2 variants identified indicating greater peptide coverage of the larger, more acidic molecule. [file 1479-5876-5-33-S1.pdf]

(a) α_2-macroglobulin

1 MGKNKLLHPS LVLLLLVLLP TDASVSGKPQ YMVLVPSLLH TETTEKGCVL
51 LSYLNETVTV SASLESVRGN RSLFTDLEAE NDVLHCVAFA VPK**SSSNEEV**
101 **MFLTVQVKGP TQEFK**KRTTV MVK**NEDSLVF VQTDKSIYKP GQTVK**FRVVS
151 MDENFHPLNE LIPLVYIQDP KGNRIAQWQS FQLEGGLK**QF SFPLSSEPFQ**
201 **GSYK**VVVQKK SGGRTEHPFT VEEFVLPK**FE VQVTVPK**IIT ILEEEMNVSV
251 CGLYTYGKPV PGHVTVSICR KYSDASDCHG EDSQAFCEKF SGQLNSHGCF
301 YQQVKTKVFQ LKRKEYEMKL HTEAQIQEEG TVVELTGRQS SEITRTITKL
351 SFVKVDSHFR **QGIPFFGQVR** LVDGKGVPIP NKVIFIRGNE ANYYSNATTD
401 EHGLVQFSIN TTNVMGTSLT VRVNYKDRSP CYGYQWVSEE HEEAHHTAYL
451 VFSPSKSFVH LEPMSHELPC GHTQTVQAHY ILNGGTLLGL KKLSFYYLIM
501 AKGGIVRTGT HGLLVKQEDM KGHFSISIPV K**SDIAPVAR**L LIYAVLPTGD
551 VIGDSAKYDV ENCLANKVDL SFSPSQSLPA SHAHLR**VTAA PQSVCALR**AV
601 DQSVLLMKPD AELSASSVYN LLPEKDLTGF PGPLNDQDDE DCINRHNVYI
651 NGITYTPVSS TNEK**DMYSFL EDMGLK**AFTN SKIRKPKMCP QLQQYEMHGP
701 EGLRVGFYES DVMGRGHARL VHVEEPHTET VRKYFPETWI WDLVVVNSAG
751 VAEVGVTVPD TITEWKAGAF CLSEDAGLGI SSTASLRAFQ PFFVELTMPY
801 SVIRGEAFTL KATVLNYLPK CIRVSVQLEA SPAFLAVPVE KEQAPHCICA
851 NGRQTVSWAV TPKSLGNVNF TVSAEALESQ ELCGTEVPSV PEHGRKDTVI
901 KPLLVEPEGL EKETTFNSLL CPSGGEVSEE LSLKLPPNVV EESARASVSV
951 LGDILGSAMQ NTQNLLQMPY GCGEQNMVLF APNIYVLDYL NETQQLTPEV
1001 KSKAIGYLNT GYQRQLNYKH YDGSYSTFGE RYGRNQGNTW LTAFVLKTFA
1051 QARAYIFIDE AHITQALIWL SQRQKDNGCF RSSGSLLNNA IKGGVEDEVT
1101 LSAYITIALL EIPLTVTHPV VRNALFCLES AWKTAQEGDH GSHVYTKALL
1151 AYAFALAGNQ DKRKEVLKSL NEEAVKKDNS VHWERPQKPK APVGHFYEPQ
1201 APSAEVEMTS YVLLAYLTAQ PAPTSEDLTS ATNIVKWITK QQNAQGGFSS
1251 TQDTVVALHA LSKYGAATFT RTGKAAQVTI QSSGTFSSKF QVDNNNRLLL
1301 QQVSLPELPG EYSMKVTGEG CVYLQTSLKY NILPEKEEFP FALGVQTLPQ
1351 TCDEPKAHTS FQISLSVSYT GSRSASNMAI VDVKMVSGFI PLKPTVKMLE
1401 RSNHVSRTEV SSNHVLIYLD KVSNQTLSLF FTVLQDVPVR DLKPAIVKVY
1451 DYYETDEFAI AEYNAPCSKD LGNA

(b) Complement C4A

1 MRLLWGLIWA SSFFTLSLQK PRLLLFSPSV VHLGVPLSVG VQLQDVPRGQ
51 VVKGSVFLRN PSRNNVPCSP KVDFTLSSER DFALLSLQVP LKDAKSCGLH
101 QLLRGPEVQL VAHSPWLKDS LSRTTNIQGI NLLFSSRRGH LFLQTDQPIY
151 NPGQRVRYRV FALDQKMRPS TDTITVMVEN SHGLRVRKKE VYMPSSIFQD
201 DFVIPDISEP GTWKISARFS DGLESNSSTQ FEVKKYVLPN FEVKITPGKP
251 YILTVPGHLD EMQLDIQARY IYGKPVQGVA YVRFGLLDED GKKTFFRGLE
301 SQTKLVNGQS HISLSKAEFQ DALEKLNMGI TDLQGLRLYV AAAIIESPGG
351 EMEEAELTSW YFVSSPFSLD LSKTKRHLVP GAPFLLQALV REMSGSPASG
401 IPVKVSATVS SPGSVPEVQD IQQNTDGSGQ VSIPIIIPQT ISELQLSVSA
451 GSPHPAIARL TVAAPPSGGP GFLSIERPDS RPPRVGDTLN LNLRAVGSGA
501 TFSHYYYMIL SRGQIVFMNR EPKRTLTSVS VFVDHHLAPS FYFVAFYYHG
551 DHPVANSLRV DVQAGACEGK LELSVDGAKQ YRNGESVKLH LETDSLALVA
601 LGALDTALYA AGSKSHKPLN MGKVFEAMNS YDLGCGPGGG DSALQVFQAA
651 GLAFSDGDQW TLSRKRLSCP KEKTTRKKRN VNFQKAINEK LGQYASPTAK
701 RCCQDGVTRL PMMRSCEQRA ARVQQPDCRE PFLSCCQFAE SLRKKSRDKG
751 QAGLQRALEI LQEEDLIDED DIPVRSFFPE NWLWRVETVD RFQILTLWLP
801 DSLTTWEIHG LSLSKTKGLC VATPVQLRVF REFHLHLRLP MSVRRFEQLE
851 LRPVLYNYLD KNLTVSVHVS PVEGLCLAGG GGLAQQVLVP AGSARPVAFS
901 VVPTAAAAVS LKVVARGSFE FPVGDAVSKV LQIEKEGAIH REELVYELNP
951 LDHRGRTLEI PGNSDPNMIP DGDFNSYVRV TASDPLDTLG SEGALSPGGV
1001 ASLLRLPRGC GEQTMIYLAP TLAASRYLDK TEQWSTLPPE TKDHAVDLIQ
1051 KGYMRIQQFR KADGSYAAWL SRDSSTWLTA FVLKVLSLAQ EQVGGSPEKL
1101 QETSNWLLSQ QQADGSFQDP CPVLDRSMQG GLVGNDETVA LTAFVTIALH
1151 HGLAVFQDEG AEPLKQRVEA SISKANSFLG EKASAGLLGA HAAAITAYAL
1201 SLTKAPVDLL GVAHNNLMAM AQETGDNLYW GSVTGSQSNA VSPTPAPRNP
1251 SDPMPQAPAL WIETTAYALL HLLLHEGKAE MADQASAWLT RQGSFQGGFR
1301 STQDTVIALD ALSAYWIASH TTEERGLNVT LSSTGRNGFK SHALQLNNRQ
1351 IRGLEEELQF SLGSKINVKV GGNSKGTLKV LRTYNVLDMK NTTCQDLQIE

1401 VTVKGHVEYT MEANEDYEDY EYDELPAKDD PDAPLQPVTP LQLFEGRRNR
1451 RRREAPK**VVE EQESR**VHYTV CIWRNGKVGL SGMAIADVTL LSGFHALRAD
1501 LEKLTSLSDR YVSHFETEGP HVLLYFDSVP TSRECVGFEA VQEVPVGLVQ
1551 PASATLYDYY NPERRCSVFY GAPSKSRLLA TLCSAEVCQC AEGKCPRQRR
1601 ALER**GLQDED GYR**MKFACYY PR**VEYGFQVK** VLREDSRAAF RLFETKITQV
1651 LHFTKDVKAA ANQMRNFLVR ASCR**LRLEPG K**EYLIMGLDG ATYDLEGHPQ
1701 YLLDSNSWIE EMPSERLCRS TRQRAACAQL NDFLQEYGTQ GCQV

(c) Apolipoprotein A-IV

Variant 1

1 MFLKAVVLTL ALVAVAGARA EVSADQVATV MWDYFSQLSN NAKEAVEHLQ
51 K**SELTQQLNA LFQDK**LGEVN TYAGDLQKKL VPFATELHER LAKDSEKLKE
101 EIGKELEELR ARLLPHANEV SQKIGDNLRE LQQRLEPYAD QLR**TQVNTQA**
151 **EQLR**RQLTPY AQRMERVLRE NADSLQASLR PHADELKAK**I DQNVEELK**GR
201 **LTPYADEFK**V K**IDQTVEELR** R**SLAPYAQDT QEK**LNHQLEG LTFQMKKNAE
251 ELKAR**ISASA EELR**QR**LAPL AEDVR**GNLKG NTEGLQKSLA ELGGHLDQQV
301 EEFRRRVEPY GENFNK**ALVQ QMEQLR**QKLG PHAGDVEGHL SFLEKDLR**DK**
351 **VNSFFSTFK**E KESQDKTLSL PELEQQQEQQ QEQQQEQVQM LAPLES

Variant 2

1 MFLKAVVLTL ALVAVAGARA EVSADQVATV MWDYFSQLSN NAKEAVEHLQ
51 KSELTQQLNA LFQDKLGEVN TYAGDLQKKL VPFATELHER LAKDSEKLKE
101 EIGKELEELR ARLLPHANEV SQKIGDNLRE LQQR**LEPYAD QLRTQVNTQA**
151 **EQLR**RQLTPY AQRMERVLRE NADSLQASLR PHADELKAK**I DQNVEELK**GR
201 LTPYADEFKV K**IDQTVEELR** RSLAPYAQDT QEKLNHQLEG LTFQMKKNAE
251 ELKAR**ISASA EELR**QR**LAPL AEDVR**GNLKG NTEGLQKSLA ELGGHLDQQV
301 EEFRRRVEPY GENFNK**ALVQ QMEQLR**QKLG PHAGDVEGHL SFLEKDLRDK
351 **VNSFFSTFK**E KESQDKTLSL PELEQQQEQQ QEQQQEQVQM LAPLES
